# Multiple roles of ribosomal antimicrobial peptides in tackling global antimicrobial resistance

**DOI:** 10.1098/rsos.211583

**Published:** 2022-01-26

**Authors:** Huy Xuan Luong, Hoa Doan Ngan, Hai Bui Thi Phuong, Thang Nguyen Quoc, Truong Thanh Tung

**Affiliations:** ^1^ Faculty of Pharmacy, PHENIKAA University, Hanoi 12116, Vietnam; ^2^ PHENIKAA Institute for Advanced Study (PIAS), PHENIKAA University, Hanoi 12116, Vietnam; ^3^ Hanoi Medical University, Hanoi, Vietnam; ^4^ Nuclear Medicine Unit, Vinmec Healthcare System, Hanoi 10000, Vietnam

**Keywords:** ribosomal antimicrobial peptides, microbial resistance, multifuntional peptides, antimicrobial agents, antibiotic management

## Abstract

In the last century, conventional antibiotics have played a significant role in global healthcare. Antibiotics support the body in controlling bacterial infection and simultaneously increase the tendency of drug resistance. Consequently, there is a severe concern regarding the regression of the antibiotic era. Despite the use of antibiotics, host defence systems are vital in fighting infectious diseases. In fact, the expression of ribosomal antimicrobial peptides (AMPs) has been crucial in the evolution of innate host defences and has been irreplaceable to date. Therefore, this valuable source is considered to have great potential in tackling the antimicrobial resistance (AMR) crisis. Furthermore, the possibility of bacterial resistance to AMPs has been intensively investigated. Here, we summarize all aspects related to the multiple applications of ribosomal AMPs and their derivatives in combating AMR.

## 1. Introduction

Antimicrobial resistance (AMR) occurs when microbial pathogens, including bacteria, viruses, fungi and parasites, change over time and no longer respond to drugs. AMR results in increased difficulties or even impossibility in treating infection and elevates the risk of spreading diseases, severe illness and death. Consequently, the cost of AMR to national economies and healthcare systems is serious, as it prolongs hospital stays, requires more expensive and intensive care, and significantly affects the productivity of patients and their caregivers [1,2]. Therefore, new antimicrobial agents are urgently required [3]. However, according to the World Health Organization (WHO), the clinical pipeline of newly developed antimicrobials is dry and insufficient to deal with the AMR challenge [4,5]. In 2020, among 26 antibiotics currently in clinical development, which are active against at least one of the priority pathogens in the WHO list, only seven candidates were classified as innovative [4]. However, even if all of these successfully pass clinical trials and enter the market, the new antibiotics will still suffer the same fate as the previous ones and soon become ineffective. In fact, the global threat of AMR needs to be tackled from many aspects at multiple levels and across different areas [6–8]. Recently, ribosomal antimicrobial peptides (AMPs) have emerged as a promising category that can effectively support many approaches for managing AMR [9–11].

**Figure 1.**
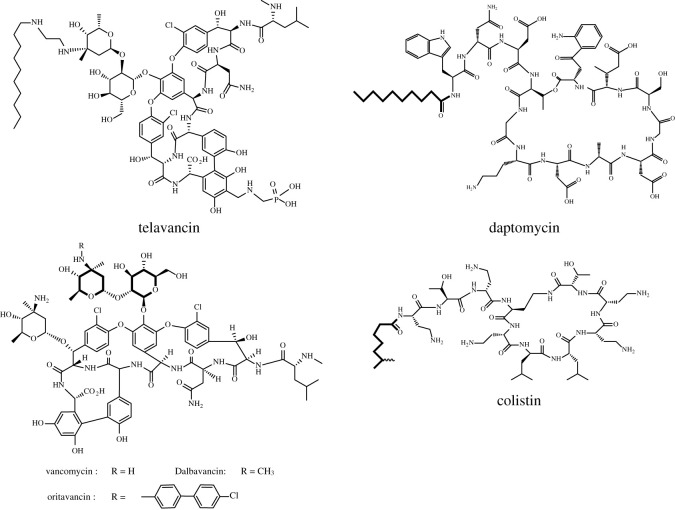
Examples of macrocyclic peptide-based antibiotic molecules.

**Figure 2.**
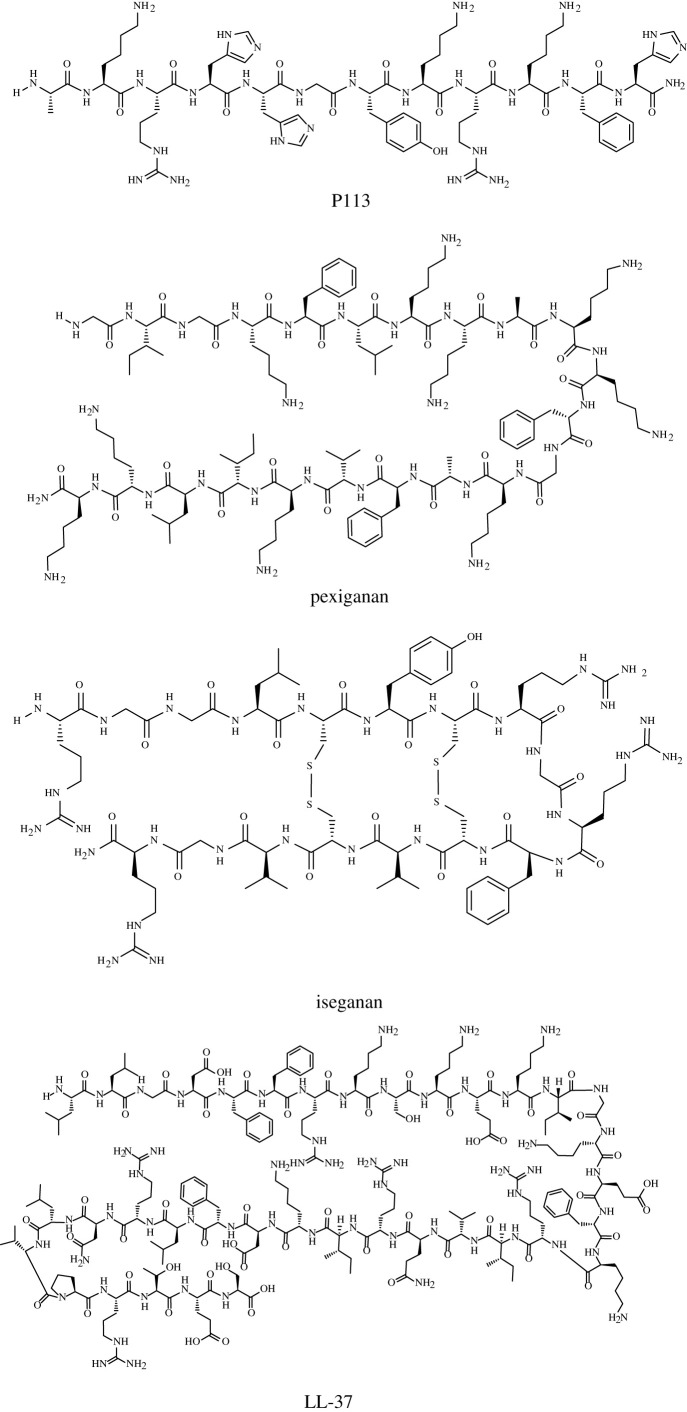
Structure of selected antimicrobial peptides in clinical trials.

Ribosomally synthesized AMPs are secreted as the first line of defence system by prokaryotes, plants and animals [11,12]. Unlike polypeptide antibiotics that have already been known and accepted for a long time [13], these AMPs have some significant differences in structural properties and biological profiles (table 1 and figures 1 and 2). These peptides mostly contain 7–50 amino acids [14–16] and usually share common structural properties, including a high content of cationic and hydrophobic residues, whereas their N-terminal is free and C-terminus is amidated. Such cationic amphipathic peptides show broad-spectrum activity, covering bacteria, fungi and viruses, as well as cancerous cells [16–19]. Interestingly, AMPs are well known for their fast and immediate killing effects [20–23]. Generally, the number of bacterial cells rapidly decreases after 15 min to 1 hour with at least 50% inhibition activity [24–27]. Furthermore, AMPs are well known for their immunomodulatory effects; on the one hand, they can recognize and activate the immune system to kill microbial pathogens, whereas on the other hand, they can suppress intense inflammation.

**Table 1.** Comparison of polypeptide antibiotics and antimicrobial peptides.

		antimicrobial peptides	polypeptide antibiotics
synthesizer		ribosomes	multifunctional enzymes
origins		bacteria, fungi, plants and animals	bacteria, fungi
structural properties	2nd structure	yes	rare
	branched structure	rare	common
	non-canonical amino acids	rare	common
	cyclization	not often (mostly via disulphide bonds)	often (results in oxazolines and thiazolines)
	other modifications	rare (mostly C-terminal amidation)	very common, including:
			*N*-methylation
			*N*-formylation
			glycosylation
			acylation
			halogenation
			hydroxylation
			oxidation and reduction
examples		LL37, magainins, indolicidins, Polybia-MP1, etc.	colistin, daptomycin, vancomycin, telavancin, etc.

Over thousands of years of evolution, ribosomal AMPs still play crucial roles in the host defence systems of living organisms. However, several drawbacks, including poor absorption, distribution, metabolism and excretion (ADME), as well as high production costs compared with other commercially available antibiotics, have hindered their clinical development. Recently, owing to the urgent need for alternative antimicrobial therapies and the modern advances in peptide synthesis, chemical modification and bioengineering techniques, AMPs have attracted considerable attention for research and development. Therefore, these multifunctional molecules can be some of the most important weapons in the battle against AMR. Starting from the diversity in the origins, structures and biological properties that lead to various therapeutic applications of AMPs, this review provides an overview and discusses their multiple roles in the management of AMR, as well as the opportunities and challenges in developing this emerging category.

## 2. Diversity of antimicrobial peptides

**Figure 3.**
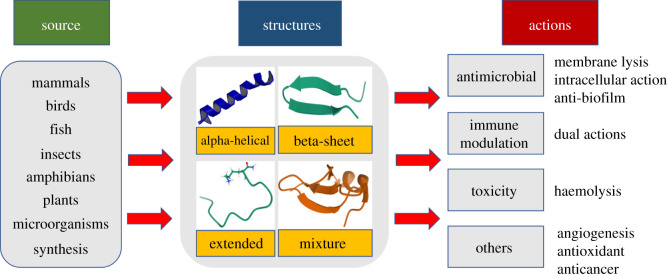
Diverse sources, structures and actions of antimicrobial peptides in nature.

As an essential component of host defence systems, thousands of AMPs have been discovered and updated into many databases and bioinformatics resources (table 2 for more details). Overall, the sources and structural conformations of AMPs as well as their multiple biological actions are rather diverse (figure 3).

**Table 2.** Some databases and bioinformatics resources of antimicrobial peptides.

databases	major contents	last updated	references
data repository of antimicrobial peptides (DRAMP)	sequences, structures, classification, physicochemical properties, activities, patent and clinical information	4 November 2021 (latest v. DRAMP 3.0)	http://dramp.cpu-bioinfor.org/ [28]
antimicrobial peptide database (APD)	sequences, structures, classification, activities, timeline, prediction, design and statistics	20 July 2021 (latest v. APD3)	https://aps.unmc.edu/AP/ [14]
database of antimicrobial activity and structure of peptides (DBAASP)	sequences, structures, classification, activities, prediction, 3D structures and statistics	latest v. DBAASP v. 3.0	https://dbaasp.org/ [29]
yet another database of antimicrobial peptides (YADAMP)	sequences, structures, classification, physicochemical properties, activities and statistics	15 October 2018	http://yadamp.unisa.it/about.aspx [30]
LAMP (a database linking antimicrobial peptides)	sequences, structures, classification, activities and statistics	10 December 2016	http://biotechlab.fudan.edu.cn/database/lamp/index.php [31]

### 2.1. Diverse sources and secondary structures of antimicrobial peptides

#### 2.1.1. Mammals, birds and fish

Defensins and cathelicidins are two large groups of AMPs isolated from mammals [32], birds [33] and fish [34,35]. Briefly, defensins are cysteine-rich peptides with a β-sheet structure and are divided into three subgroups: α-defensins, β-defensins and θ-defensins. In contrast to defensins, which are characterized and stabilized by three disulphide bridges, cathelicidins have a wide range of structures, number of residues and sequential differences. However, most of the known cathelicidins are linear with an α-helical conformation, including LL-37 [36], PMAP-36 [37,38] and CRAMP [39].

#### 2.1.2. Amphibians

Currently, amphibians account for the largest proportion of AMPs found in nature, most of which are derived from skin secretions [22,40]. For example, 23 novel AMP sequences were discovered from a wild amphibian, *Hypsiboas pulchellus,* in Argentina [41]. Many other AMPs can be counted, such as magainins from the African frog *Xenopus laevis* [42], brevinins and esculentin found in *Rana* species [43,44] and dermaseptin from the frog genus *Phyllomedusa* [45,46]. Despite large differences in size and sequence, these AMPs in most cases, still adopt an α-helix conformation in membrane-mimicking solutions to form a cationic amphiphilic helical structure [41].

#### 2.1.3. Insects

Several AMP families have been reported to possess various secondary structures. For example, cecropins [47], lasioglossins [48], melittin [49] and Polybia-MP1 [50–52] form α-helical regions. Insect defensins form β-sheet conformations or proline/glycine-rich peptides [53], for example, drosocin [54], lebocins and attacin [55] show extended structures.

#### 2.1.4. Plants

Many reports have indicated that plants produce different types of bioactive compounds to defend against the invasion of fungi, bacteria and insects [56]. Thus, AMPs have also been found in many plant components, such as fruits, flowers, leaves and stems [57,58], most of which contain cysteine residues and form disulphide bonds [57–59]. Recently, short disulphide-free AMPs were also found in green coconut water and proven to be multifunctional peptides without any sign of cytotoxicity to human cells [60–63].

#### 2.1.5. Microorganisms

Bacteria and fungi produce a wide range of antimicrobial agents. However, non-ribosomal peptide antibiotics such as polymyxins, vancomycin or teixobactin are generally referred as polypeptide antibiotics, whereas the term ‘AMPs’ is more commonly used for ribosomal antibiotics. Hence, some well-known AMPs include nisin, microsin and pediocin from bacteria [64–66] and plectasin from fungi [67].

#### 2.1.6. Synthetic sources

In addition to ribosomally synthesized molecules isolated from nature, several artificial AMPs have been created [27,68–70]. These peptides can be designed and synthesized based on the structure–activity relationships (SARs) of natural antimicrobial agents, aimed at improving one or more pharmacological properties [71,72]. Moreover, owing to the vast number of natural peptides with diverse lengths, structures and mechanisms of action, it is difficult to obtain reliable and complete SAR data. Therefore, the de novo design of AMPs based on general structural requirements was applied, and promising data were obtained [69,73,74].

### 2.2. Diverse biological effects of AMPs

Ribosomally synthesized AMPs have been demonstrated to be active against various types of pathogens, including bacteria, fungi, viruses, protozoa and cancer cells [17,75,76]. The most common mode of action of AMPs is membrane lysis via pore formation, leading to bacterial cell death [10,77,78]. The net positive charge, which is provided by cationic residues (arginine and lysine), the free amino group at the N-terminus, and the charge distribution are important for initial binding to the bacterial membrane [79]. Notably, amidation of the C-terminus benefits the increased net charge as well as structural stability and enhances membrane activity [48,80–82]. Next, the hydrophobic interaction between multiple hydrophobic residues and lipid bilayers can induce membrane hyperpolarization [83,84], membrane permeation and destruction through various models, including the barrel stave, carpet model, membrane thinning or thickening, electroporation, and toroidal and disordered toroidal pores [10,32,76]. This mechanism of action is selective for the negatively charged outer bacterial membrane over zwitterionic mammalian membranes [85,86]. Furthermore, in contrast to the lack of cholesterol in the bacterial membrane, the presence of cholesterol in eukaryotic cell membranes was demonstrated to reduce the interaction with AMPs and to suppress the disruption of lipid bilayers [87–91]. Furthermore, after entering bacterial cells without membrane disruption, other mechanisms of action have also been explored, as AMPs have been reported to inhibit some intracellular functions [17,92–94]. They can interact with negatively charged nucleic acids to interfere with their synthesis, replication and translation [95–99]. AMPs also target the biosynthesis [100–103], folding [104–106] and enzymatic activity [107–109] of proteins as well as metabolic processes [110–112] to achieve bacterial cell killing. Interestingly, AMPs have recently been reported to sequester and restrict the access of essential metals in invading pathogens [113].

Regarding immune system modulation, many AMPs are known to recruit and activate other immune components to clear infection. However, they can also act as suppressors when the inflammation becomes too strong by neutralizing bacterial products such as the endotoxin lipopolysaccharide (LPS) and lipoteichoic acid (LTA), and can control the Toll-like receptor (TLR) response [114,115]. Thus, AMPs are considered safer than conventional antibiotics. Consequently, several AMPs are currently in clinical trials for the management of septic shock [116,117].

Moreover, AMPs can also play active roles in wound healing [17,118–120] via antimicrobial activity as well as modulation of cytokine production, cell migration, proliferation, collagen synthesis and in some cases, angiogenesis [120–123]. Notably, wound healing is not unique to mammals and can also be observed in other species, such as fruit flies [124,125] and amphibians [126,127]. Furthermore, AMPs with antioxidant potential have also been also found in fishes [128–130], frogs [131,132] and molluscs [133], thus demonstrating their protective effect against reactive oxygen species (ROS) in anti-ageing strategies. In the near future, AMPs are promising candidates for fighting resistant bacteria as well as promoting wound healing and skin regeneration [118,134,135].

### 2.3. Diverse therapeutic applications of antimicrobial peptides

**Figure 4.**
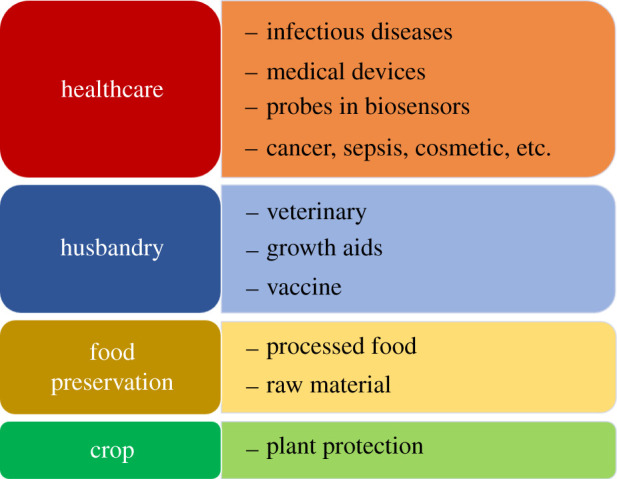
The diverse applications of antimicrobial peptides.

The current applications of AMPs are divided into four main groups: human healthcare, husbandry, food preservation and plant protection (figure 4). The majority of research effort to date has been focused on developing new therapies for various aspects of human health, such as infectious diseases [9,10,136], medical devices [17,137], cosmetology [118,135,138,139], cancer [51,140–144] and septic shock [114,145–147]. The broad-spectrum, along with the fast and selective actions of antimicrobial peptides can also benefit the development of biosensors that can rapidly detect pathogenic threats or monitor bacterial contaminations [148–150].

Many natural AMPs have been exploited for livestock, including veterinary medicines such as nisin. The FDA has approved nisin for dairy animals suffering from mastitis [151], as an adjuvant for new vaccine formulations [9,152,153] and as a growth aid [154,155]. Moreover, after nisin was the first bacteriocin approved for use as a food additive with code E234 [156–158], AMPs are now considered a new generation of food preservatives in both processed foods and their raw materials [159–161]. Interestingly, several studies have examined the potential of AMPs in plant protection, and some of these are introduced in table 3 [59,69,162–164].

**Table 3.** Sequence of some potential antimicrobial peptides for plant protection.

peptide	sequence	references
SP1-D	RKKRLKLLKRLV-NH_2_	[69]
SP7-D	LLIKFLKRFIKH-NH_2_	
SP10-D	LRFLKKILKHLF-NH_2_	
SP13-D	KRRLIARILRLAARALVKKR-NH_2_	
BP100	KKLFKKILKYL-NH2	[162]
BP134	KKLFKKILKYL-OH	[163]
BP203	KKLFKKILKYL-KKLFKKILKYL-OH	
BP209	G-KKLFKKILKYL-AGPA-GIGKFLHSAK-OH	
BP210	S-KKLFKKILKYL-AGPA-GIGKFLHSAK-OH	

Conventional antibiotics, as well as other antifungal, antiviral and antiparasitic drugs, are becoming increasingly ineffective as AMR has spread globally among humans, animals and in the environment [165]. This means that it is more challenging to manage infectious diseases and that the number of relevant deaths will increase, especially in patients with high-risk medical conditions, and undergoing treatments such as cancer chemotherapy, organ transplantation and other surgical indications [166,167]. Ribosomal AMPs with diverse mechanisms of action have become one of the most promising alternative antimicrobial agents to address the crisis of antibiotic resistance. Accordingly, they have been demonstrated to be active against a wide range of multi-drug-resistant pathogens, including those in the list of WHO priority pathogens for the research and development of new antibiotics (table 4 for some recent AMPs demonstrated to be active against antibiotic-resistant bacteria).

**Table 4.** Recent antimicrobial peptides that active against some drug-resistant species.

priority^a^	pathogens^a^	antibiotic resistance^a^	antimicrobial peptides
critical	*Acinetobacter baumannii*	carbapenem-resistant	Cec4 [168], Ω76 [169], ZY4 [170], Hp1404 [171], TP4 derivatives dC4 and dN4 [172], AMPR-11 [114].
	*Pseudomonas aeruginosa*	carbapenem-resistant	ZY4 [170], P5 [173], AMPR-11 [114], ΔM2 [174], Ci-MAM-A24 [175].
	*Enterobacteriaceae*	carbapenem-resistant, ESBL-producing	AMPR-11 [114], DRGN-6 [176], ΔM2 [174], A-thanatin [177], Arenicin-3 [178], AA139 [178], Ci-MAM-A24 [175].
high	*Enterococcus faecium*	vancomycin-resistant	Ci-MAM-A24 [175], Bip-P-113 [179], SLAY-P1 [180], Nisin [181], Lacticin 3147 [181].
	*Staphylococcus aureus*	methicillin-resistant, vancomycin-intermediate and resistant	Ci-MAM-A24 [175], Nisin [181], Lacticin 3147 [181], WR12 [182], D-IK8 [182]. Melittin [183]
	*Helicobacter pylori*	clarithromycin-resistant	Cbf-K_16_ [184], CRAMP [185], LL-37 [185], sLL-37 [185], TP4 [186]

^a^According to the list of WHO priority pathogens [187,188].

### 2.4. Resistance strategies against antimicrobial peptides

Broad-spectrum antibiotics are usually more susceptible to drug resistance. Therefore, it is possible that AMPs with notably broad-spectrum activity can suffer from microbial resistance compared with conventional antibiotics. Such resistance mechanisms have been reported for both types of bacterial species [189–191] and can be classified into four major groups:

— Membrane modification: bacteria can decrease the attraction and insertion of AMPs into their membrane by reducing the overall negative charges (such as by *D*-alanylation of teichoic acid [192], addition of 4-aminoarabionse to lipid A [193]), and enhancing rigidity (for example, by biofilm formation [194] or lipid A acylation [195,196]).
— Efflux pumps: export of antimicrobial agents out of the cell is an important strategy to remove several conventional antibiotics as well as antimicrobial peptides, such as LL-37, defensins and CRAMP [197,198].
— Proteolytic degradation: bacteria use proteases such as metalloproteinase [199–201], cysteine protease [202,203], or the omptin family of aspartate proteases [204,205] to break AMPs and avoid their killing action. For example, aureolysin is a zinc metalloprotease belonging to the thermolysin family that cleaves peptide bonds between Leu_31_-Val_32_, Arg_23_-Ile_24_, and Arg_19_-Ile_20_ in LL-37 [201,206]. Hence, it is suggested that *S. aureus* strains with significant secretion of this proteinase are less susceptible than those that do not express aureolysin activity [201].
— Sequestration: bacteria block AMPs from outside the cell, thus preventing them from reaching the bacterial cell membrane [207–209].

### 2.5. Low propensity to induce resistance and cross-resistance to antimicrobial peptides

Recently, a polypeptide antibiotic, colistin and an antimicrobial peptide, ZY4, were evaluated for their propensity to develop resistance in several *P. aeruginosa* and *A. baumannii* strains [170]. Accordingly, bacteria were exposed to ZY4 or colistin in the presence of sub-inhibitory concentrations. After the first 20 passages, the minimum inhibitory concentrations (MICs) of colistin steadily increased, whereas no appreciable change was observed in the antimicrobial activity of ZY4. This difference became more significant after 60 passages, by which the MICs of colistin increased by 16–25 times compared with those of ZY4, which increased by 4.0–4.5 times [170]. Further investigation suggested that there was no observed cross-resistance between ZY4 and two similar antimicrobial peptides, ZY13 [210] and LZ1 [211] with other antibiotics, including colistin, tobramycin and levofloxacin [170].

In a different experimental approach, the integrated evolutionary analysis of two antimicrobial peptides, Tachyplesin II and Cecropin P1, confirmed the relatively low frequency of resistance through point mutations and gene amplification [212]. Furthermore, an investigation of the AMPs and antibiotic resistance genes in the human gut microbiota revealed that these two kinds of genes are different in adapting to new bacterial hosts. Consequently, the transfer tendency between the members of antimicrobial peptide resistance genes is less frequent than the others [213].

Although bacteria can develop resistance to antimicrobial peptides, it is suggested that the diversity in their mechanisms of action can provide an effective therapy to control bacterial growth [214]. For example, bacteria can increase electrostatic repulsion to defend themselves against some high cationic charge antimicrobial peptides; however, this strategy may not be practical for peptides with low net charge or multiple anionic residues (e.g. Polybia-MP1 [72]). Moreover, there is a minority group of AMPs with an anionic net charge [215–217] that can avoid this membrane modification. In the case of biofilms or capsule formation, there are always other AMPs with anti-biofilm properties [218] or the ability to destroy the protective capsule [219]. It is thus possible that the activity of an individual antimicrobial peptide is significantly reduced owing to a specific resistance mechanism in microbial pathogens; however, using a combination of AMPs or of AMPs with current antibiotics can promote synergistic action and overcome resistance [17,22]. It is also worth mentioning that innate host defence systems usually contain multiple types of AMPs with some differences in structure and function. Furthermore, the rapid action of AMPs [17] and the energy costs for developing multiple defence capacities are major obstacles in the proliferation and growth of microbial pathogens, thus limiting their resistance to antimicrobial peptide therapies.

### 2.6. AMPs and current therapies for antimicrobial resistance

**Figure 5.**
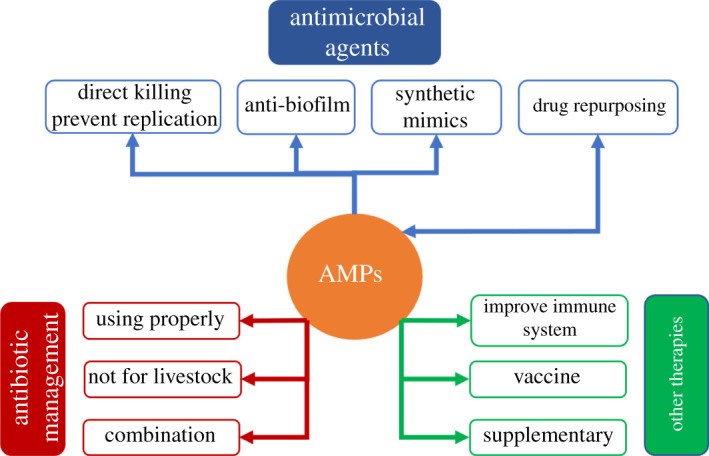
Diagram illustrating the correlation of antimicrobial peptides and the current therapies in antibiotic resistance management.

AMR is a complicated problem that requires various strategic solutions; fortunately, AMPs are closely related to most of resistance mechanisms (see the summary in figure 5).

#### 2.6.1. Antibiotics management

One of the main drivers of the multi-drug resistance crisis is the misuse and overuse of antimicrobials. Therefore, one of the important objectives is to promote the proper use of current drugs, which AMPs can support through several approaches such as replacing or at least reducing the traditional doses of antimicrobials, for example, by increasing antimicrobial peptide use for topical applications and in combination therapy [17,138,220]. Notably, according to the Food and Drug Administration (FDA), approximately 80% of all antibiotics were sold for use on livestock farms in 2014—the same year that the WHO published its first ever report on global AMR [221]. Accordingly, medically important antibiotics are limited or even banned in animal food in the USA and other countries [222]. Therefore, AMPs can be considered as one of the most promising antibiotic alternatives for both human and animal use.

#### 2.6.2. New antimicrobial agents

High rates of resistance against frequently used antimicrobials have been observed everywhere, indicating that the world is running out of effective drugs for infectious disease. In drug development, ribosomal AMPs and their derivatives can serve as alternative antimicrobial agents, anti-biofilm agents or even both [21,223]. In fact, many AMPs, such as Histatin [224], Plectasin [67], Omiganan [10], IMX942 [225], Iseganan [9], LL-37 [9] and P113 [9], are currently in pre-clinical or clinical studies for various anti-infectious applications.

Additionally, efforts are underway in drug repurposing (or drug repositioning) to address the absence of new antimicrobial agents and limit the risk of failure and high costs required for development [226,227]. Thus, AMP development and drug repurposing are complementary in the fight against antibiotic-resistant bacteria. Moreover, AMPs can inspire the investigation of possible drug repurposing for antimicrobial discovery. For example, glatiramer acetate (GA), also referred to as COP-1, is a popular and safe treatment for multiple sclerosis. The structure of GA is similar to the cationic amphiphilic property of standard antimicrobial peptides, thus leading to the investigation of its antibacterial activity. GA was thus demonstrated to be active against both Gram-negative and Gram-positive bacterial species, including *Pseudomonas aeruginosa*, *Escherichia coli*, *Acinetobacter baumannii and Staphylococcus aureus* [228–231]. In particular, it displayed higher potency towards Gram-negative *Pseudomonas aeruginosa* compared with the human antimicrobial peptide LL-37 [228].

**Figure 6.**
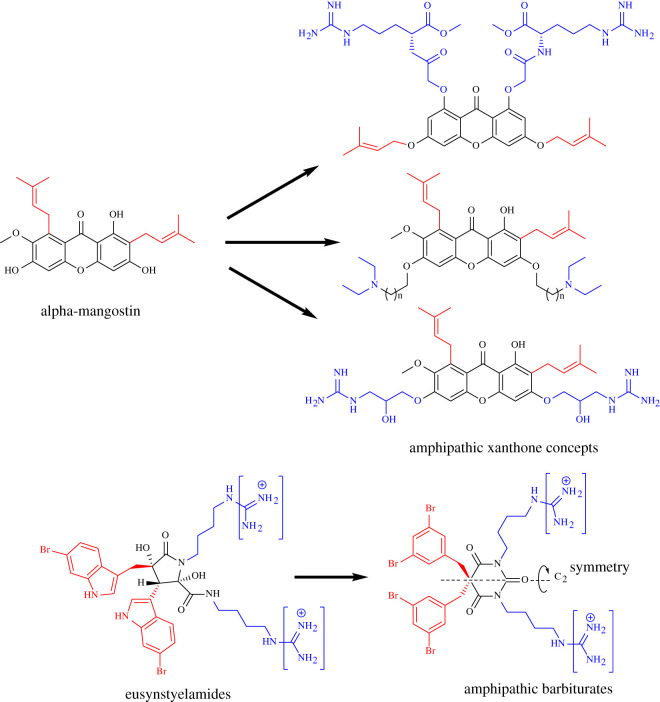
Synthetic mimics of cationic antimicrobial peptides.

Notably, synthetic mimics of cationic AMPs (CAMPs) have been demonstrated to generate promising compounds for further development of new anti-infectious diseases [232–235]. Based on the common pharmacophore of short antimicrobial peptides, this strategy provides an attractive option that can avoid protease degradation and salt sensitivity, whereas large-scale production can be simple and cheap [236,237]. Alpha-mangostin, a xanthone extracted from *Garcinia mangostana*, showed potent and rapid antibacterial activity against Gram-positive species [238]. However, this compound has major disadvantages for clinical applications, including unsatisfactory cytotoxicity and poor aqueous solubility. Therefore, a series of amphipathic xanthones were designed as membrane-targeting antimicrobial peptidomimetics [237] (figure 6). Through systematic modifications of the cationic and hydrophobic moieties, some optimized compounds have displayed excellent and fast antibacterial activity against Gram-positive bacteria, including vancomycin-resistant enterococci and methicillin-resistant *S. aureus*, with higher selectivity and low propensity to develop resistance [239–241]. In another approach, inspired by the pharmacophore model CAMPs and marine antimicrobials, eusynstyelamides, a novel series of amphipathic barbiturates were designed with two cationic groups and two lipophilic side chains (figure 6). The obtained data suggested potent lead peptidomimetic compounds with broad-spectrum *in vitro* activity against 30 multi-drug resistant clinical isolates and exhibited promising *in vivo* efficacy in a mouse model infected with *Klebsiella pneumoniae* and *E. coli* [242].

#### 2.6.3. Other therapies

In addition to their potential as new antibacterial agents, the benefits of AMPs have also been exploited to improve human and animal health, mainly by improving the immune system and intestinal morphology [243–247]. Nonetheless, most of these applications are currently in livestock and veterinary medicine [155].

## 3. Advantages, limitations and solutions

As described above, natural AMPs have various advantages in replacing traditional antibiotics. Broad-spectrum activity, diversity of mechanisms, fast action, lower risk of resistance and low propensity to develop toxicity are some of the most notable advantages [248]. Interestingly, in contrast to the high toxicity of available polypeptide antibiotics, many recent *in vivo* studies have reported that AMPs are safe for animal models [70,223,249]. Notably, by testing the lysis of human renal proximal tubular epithelial cells (HRPTEC) *in vitro* and applying some sequence modifications, the possibility of reduced renal function can be avoided both *in vitro* and *in vivo* [70]. Furthermore, despite cytotoxicity to human red blood cells *in vitro*, there were no significant anaemia symptoms reported in animal models. In fact, even melittin, a well-known antimicrobial peptide with high haemolytic properties (EC_50_ < 1 µM), was confirmed to be safe for use in mice [250].

It should be noted that AMPs also have several limitations, including moderate antimicrobial activity, large size and poor *in vivo* bioavailability [17,248]. Thus, it is essential to find practical solutions to overcome these. To date, numerous strategies have been proposed including combination therapies [220,251–253], chemical modification approaches [254–258], optimization of peptide synthesis and structure [259,260] and formulation strategies [261–263]. A summary of some factors in the development of small molecules and AMPs as new antimicrobial drugs is presented in table 5.

**Table 5.** Summary of the advantages, limitations and solutions of small molecules and AMPs in the development of novel antimicrobial agents.

	small antibiotic molecules	natural AMPs
advantages	lower cost	broad spectrum
	stable	various mechanisms for each
	good permeability	fast action
	good oral bioavailability	lower propensity to develop toxicity or resistance
limitations	narrow spectrum	high cost
	mostly one mechanism for each	unstable
	higher propensity to resistance	low permeability
	high risk of drug–drug interaction	sensitive to environmental changes (pH, salts, fluids, …)
solutions	management of undesirable outcomes	optimize the synthesis process
	combination therapy	
	biological and chemical strategies	
	choose proper routes of administration	

## 4. Production of commercial antimicrobial peptides

There are currently two major technologies for producing commercial peptides, including chemical and microbial production, each with different strengths and weaknesses. The chemical method requires less time to develop and is easier for purification. However, its disadvantages include high production costs, difficulty in synthesizing long peptide sequences, and the use of environmentally unfriendly solvents [264,265]. Recombinant production can overcome the weakness of its chemical counterpart; however, it is more complex, labour-consuming, has difficult purification, and is greatly restricted by natural amino acids and vectors used [266,267]. Moreover, the high expression of AMPs could induce a killing effect on yeast and bacteria, thus resulting in a low yield and endotoxin release [268]. Therefore, it is suggested that chemical technology is more suitable for human use with high purity requirements, especially for producing AMPs with non-canonical amino acids and other chemical modifications. In addition, recombinant technology is widely applied for veterinary, animal growth aid and plant protection owing to the balance between cost and efficacy [243,267,269].

## 5. Conclusion

The emergence and spread of AMR will be accelerated without effective tools for the adequate treatment of infectious diseases and antimicrobial stewardship. This review provides an overview of the potential and recent advances in the research and development of AMPs to resolve the current global crisis of antimicrobial drug resistance. The diverse origins and mechanisms of action of natural AMPs can be favourable for developing alternative antimicrobial agents [9,10,270] and can provide widespread support for many other aspects in the management of AMR. In addition to latest findings that suggested a low propensity to develop resistance and toxicity, AMPs can be some of the most potent weapons in the war against resistant microbial pathogens.

## Data accessibility

This article does not contain any additional data.
